# Strain Analysis of Left Ventricular Function in the Association of Hypertrophic Cardiomyopathy and Systemic Arterial Hypertension

**DOI:** 10.5935/abc.20190176

**Published:** 2019-10

**Authors:** Thereza Cristina Pereira Gil, Marcia Bueno Castier, Alyne Freitas Pereira Gondar, Ana Ferreira Sales, Marceli de Oliveira Santos, Fernanda Cristina da Silva de Lima, Ricardo Mourilhe-Rocha

**Affiliations:** 1 Universidade do Estado do Rio de Janeiro, Rio de Janeiro, RJ - Brazil; 2 Instituto Nacional de Câncer, Rio de Janeiro, RJ - Brazil; 3 Instituto Biomédico - Universidade Federal Fluminense, Niterói, RJ - Brazil

**Keywords:** Ventricular Function, Left, Cardiomyopathy, Hypertrophic, Hypertension, Strain, Heart Failure

## Abstract

**Background:**

Hypertrophic cardiomyopathy (HCM) is the most common heart disease of genetic origin in the world population, with a prevalence of at least 1/500. The association with systemic arterial hypertension (SAH) is not uncommon, as it affects approximately 25% of the world population. Most studies aim at the differential diagnosis between these diseases, but little is known about the magnitude of this association.

**Objective:**

To compare left ventricular global longitudinal strain (GLS) in HCM patients with and without associated SAH.

**Methods:**

Retrospective cross-sectional study that included 45 patients with HCM and preserved ejection fraction, with diagnosis confirmed by magnetic resonance imaging, including 14 hypertensive patients. Transthoracic echocardiography was performed, with emphasis on left ventricular myocardial strain analysis using GLS. In this study, p < 0.05 was considered statistically significant.

**Results:**

Left ventricular strain was significantly lower in hypertensive individuals compared to normotensive individuals (-10.29 ± 2.46 vs. -12.35% ± 3.55%, p = 0.0303), indicating greater impairment of ventricular function in that group. Mean age was also significantly higher in hypertensive patients (56.1 ± 13.9 vs. 40.2 ± 12.7 years, p = 0.0001). Diastolic dysfunction was better characterized in hypertensive patients (p = 0.0242).

**Conclusion:**

Myocardial strain was significantly lower in the group of patients with HCM and SAH, suggesting greater impairment of ventricular function. This finding may be related to a worse prognosis with early evolution to heart failure. Prospective studies are required to confirm this hypothesis.

## Introduction

The first cases of hypertrophic cardiomyopathy (HCM) were published in the 1860s, in France, related to left ventricular outflow tract obstruction.^1^ In 1957, Brock authored the first report based on hemodynamic, surgical and necropsy findings, describing the disease as subvalvar aortic stenosis with functional left ventricular obstruction, which may be related to systemic arterial hypertension (SAH).^2^ In 1958, Teare published the first histopathological description of obstructive HCM.^3^ The nonobstructive form was described by Braunwauld et al. in 1963, and confirmed by subsequent studies.^4,5^

HCM is currently defined as the primary myocardial disease of genetic origin with the highest prevalence in the world population (at least 1/500), regardless of ethnicity, sex or age, being the leading cause of sudden death in young people.^5,6^ It results from the mutation of one or more sarcomere genes, presenting significant diversity in phenotypic expression and clinical course. It is characterized by an increase in ventricular wall thickness that cannot be explained by an overload condition alone. The non-obstructive form of the disease is more frequent.^5,7^

SAH affects approximately 25% of the world population. Data from VIGITEL (2006-2014) and the World Health Organization confirm this prevalence in the Brazilian population.^8,9^ Due to the high prevalence of SAH, the association of SAH and HCM is not uncommon.

Differential diagnosis between HCM and hypertensive heart disease has been a challenge in many situations where the phenotypic expression of these diseases is similar.^10^ In this context, echocardiography has become an important tool, especially with the advent of new technologies, such as myocardial strain analysis, which assists differential diagnosis. Besides, global longitudinal strain (GLS) analysis detects early abnormalities in ventricular function before impairment of ejection fraction.^11,12^ The purpose of this study was to compare left ventricular GLS in HCM patients with and without SAH, and to assess the impact of this association on ventricular function.

## Methods

### Study participants

A retrospective cross-sectional study was conducted between September 2014 and April 2016 in patients followed up at the cardiology outpatient clinic of Hospital Universitário Pedro Ernesto - UERJ - diagnosed with HCM. This study was approved by the Research Ethics Committee with Certificate of Presentation for Ethics Appreciation (CAAE) number 23561113.2.0000.5259, and it is according to the Helsinki Declaration of 1975 updated in 2013. All patients who accepted to participate in the study have read and signed an informed consent form.

Inclusion criteria were: diagnosis of HCM confirmed by magnetic resonance imaging (MRI), age over 18 years, preserved left ventricular ejection fraction (EF) (>55%), no interventions for septal reduction and no pacemaker or defibrillator. Patients with atrial fibrillation and known coronary artery disease were excluded.

**Figure 1:**
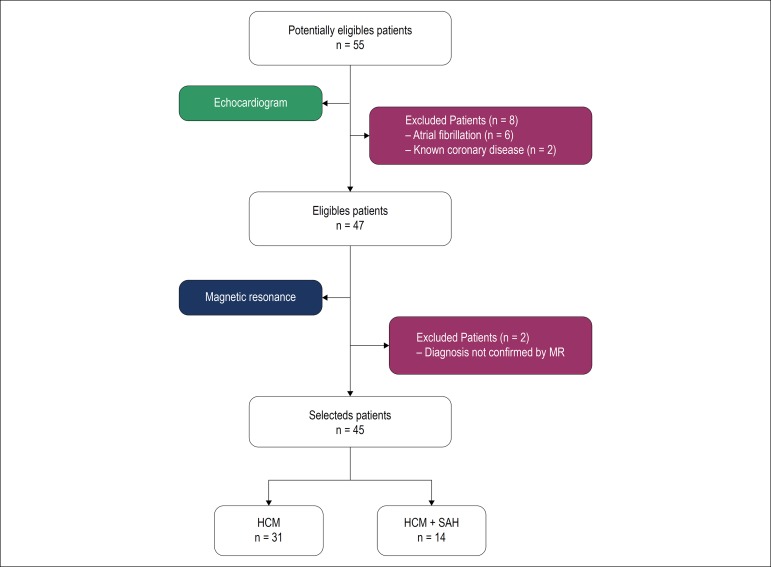
Flowchart of patient selection. MR: magnetic resonance; HCM: hypertrophic cardiomyopathy; SAH: systemic arterial hypertension.

Diagnostic confirmation by MRI with gadolinium was based on the distribution of hypertrophy and late enhancement pattern.^13^ The convenience sample included 45 patients, including 22 (48.9%) males with mean age of 45.1 ± 13.9 years. In this group, 14 (31.1%) had hypertension previously diagnosed according to the Brazilian guidelines for SAH.^8^ The flowchart with the selection of patients is shown in Figure 1.

### Echocardiographic analysis

Transthoracic echocardiographic test was performed by an experienced echocardiographer on a Philips® iE33 Matrix equipment using 3-1 MHz matrix transducer. One-dimensional, two-dimensional and Doppler echocardiography analysis was performed following the recommendations of the American Society of Echocardiography.^14^

**Figure 2:**
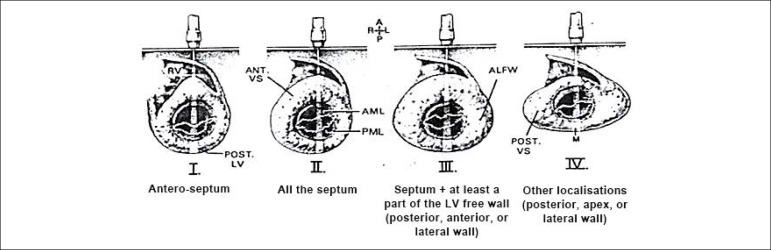
Phenotypic classification originally described by Maron. Type I: hypertrophy involving the basal septum; Type II: hypertrophy involving the entire septum; Type III: hypertrophy involving the septum and at least part of the left ventricular free wall (posterior, anterior or lateral); Type IV: other isolated locations (posterior, apical or lateral).

The echocardiographic classification of Maron et al.,^15^ which divides hypertrophy into types I, II, III, and IV (Figure 2) was used to define the type of left ventricular hypertrophy. The obstructive pattern was considered for left ventricular outflow tract gradients greater than 30 mmHg, measured by continuous Doppler, at rest and after Valsalva maneuver.^5^ The approach of diastolic function and ventricular filling pressures followed the recommendations of the American Society of Echocardiography for patients with HCM.^16^

Maron BJ. et al.^15^

In the myocardial strain analysis, we used speckle tracking. Strain is calculated for each left ventricular segment as the relative mean strain between two speckles. As a measure of strain, it is expressed in negative percentages (-%); the closer to 0, the lower the strain. Values lower than -18% were considered normal strain. Only GLS was analyzed because it is more widely used and considered a robust index for clinical studies. Also, GLS is the first to be impaired in most heart diseases, including HCM, when ejection fraction is still preserved.^12^

**Figure 3:**
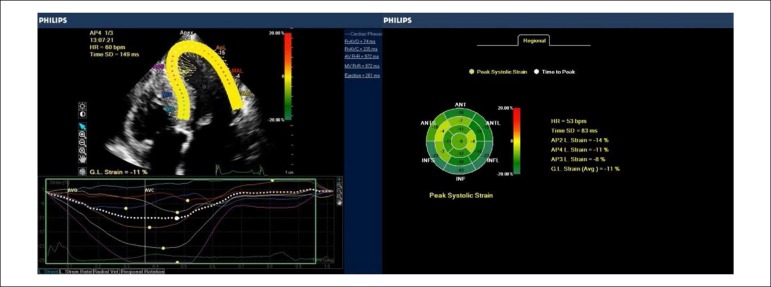
Global longitudinal strain systolic peak curves in the four-chamber apical section (left) and the parametric image of the left ventricle in the bulls-eye (right) in a patient with HCM and hypertension.

Echocardiographic protocol for GLS included 4-chamber, 3-chamber and 2-chamber apical views. GLS analysis was processed offline using the software Philips® QLab 9.0. These results were translated into curves, one for each ventricular segment, and the overview, with the quantification of velocities, was expressed on a bull’s eye map, exemplified in Figure 3.

Echocardiographic scans were stored and the images revised. Examiner and reviewer are the authors of the study. Strain analysis was repeated by the reviewer on all scans. Intraobserver and interobserver variability was evaluated using the coefficient of variation (CV = 100 (s/x) (%)). We obtained good agreement and the coefficients were considered low (<10%).

### Statistical analysis

The collected data were entered in a Microsoft Excel™ spreadsheet, and later analyzed in R Studio, version 1.0.143. Continuous variable distributions were expressed using mean and standard deviation as measures of central tendency and dispersion for each of the groups analyzed. To assess whether there was a difference between the groups, unpaired Student’s *t*-test was used after Levene’s test for equality of variances. For categorical variables, the nonparametric approach was chosen, where the difference between proportions was evaluated by the *X^2^* test (with Yates correction) and Fisher’s exact test. In cases where there were more than two categories, the Kruskal-Wallis test was used. In this study, p < 0.05 was considered statistically significant.

## Results

**Figure 4:**
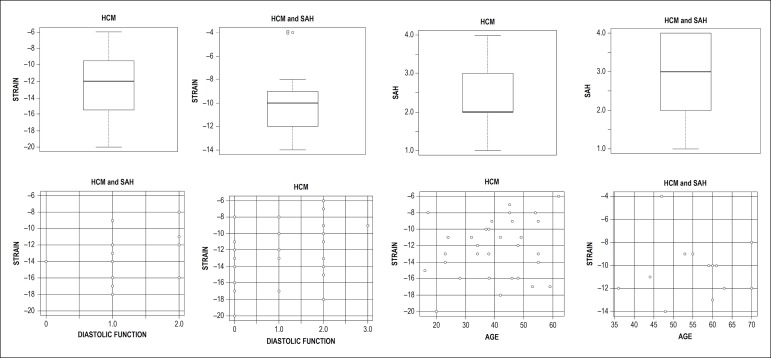
Graphs showing the main results of patients with hypertrophic cardiomyopathy (HCM) with and without associated systemic arterial hypertension (SAH). The analyzed STRAIN is the global longitudinal strain. LVH refers to the types of left ventricular hypertrophy (I, II, III, and IV) and diastolic function refers to types I, II, and III.

Of 55 initially eligible patients, 10 were excluded: 6 by atrial fibrillation, which impairs GLS analysis, 2 by known coronary artery disease, which also interferes with strain analysis, and 2 whose HCM diagnosis had not been confirmed by MRI. The patients’ general characteristics are shown in Table 1. Figure 4 shows a set of charts with the main results. Mean age was higher in the hypertensive group, and body mass index (BMI) and mean systolic and diastolic pressures were higher in this group. No significant differences were observed regarding gender and functional class between the groups.

**Table 1:** Characteristics of HCM patients in the different groups

Variables	Normotensive patients (n = 31)		Hypertensive patients (n = 14)	p
Age (years)	40.16 ± 12.73		56.14 ± 13.87	0.0001
Male sex	15 (48%)		7 (50%)	0.9323
BMI (kg/m^2^)	25.6 ± 3.97		29.2 ± 2.93	0.0045
SBP (mmHg)	113 ± 12		128 ± 12	0.0004
DBP (mmHg)	71 ± 9		81 ± 9	0.0027
**Functional Class (NYHA)**				**0.1110**
I	12 (38.7%)		2 (14.3%)	
II	19 (61.29%)		11 (78.57%)	
III	0 (0%)		1 (7.14%)	
**Hypertrophy type**				**0.1492**
I	5 (16.1%)		2 (14.3%)	
II	12 (38.7%)		2 (14.3%)	
III	12 (38.7%)		6 (42.9%)	
IV	2 (6.5%)		4 (28.5%)	
LVOT obstruction	9 (29%)		6 (43%)	0.5133
**Medications**				
Beta-blocker	22 (70%)		12 (86%)	0.4578
ACEI	2 (6.45%)		4 (28.57%)	0.0651
ARB	1 (3.23%)		11 (78.57%)	< 0.0001
Calcium antagonist	2 (6.45%)		5 (35.71%)	0.0226
Nitrate	1 (3.23%)		1 (7.14%)	0.0503
Hydralazine	0 (0%)		1 (7.14%)	0.3111
Diuretics	0 (0%)		8 (57.14%)	< 0.0001

Values expressed as mean±standard deviation or proportion, as indicated. BMI: body mass index; SBP: systolic blood pressure; DBP: diastolic blood pressure; NYHA: New York Heart Association; ACEI: angiotensin converting enzyme inhibitor, ARB: angiotensin receptor blocker.

Regarding the echocardiographic findings, there was less strain in hypertensive patients (-10.29% ± 2.46) than in normotensive patients (-12.35% ± 3.55), indicating greater impairment of ventricular function in this group (p = 0.0303). Although all patients had preserved EF, mean left ventricular systolic diameter (LVSD) was higher in hypertensive patients, but still within normal limits.

Diastolic dysfunction was more evident in hypertensive patients (p = 0.0242), with a lower number of undetermined cases. In hypertensive patients, longer isovolumetric relaxation time (IVRT), lower E/A ratio in mitral flow, as well as a lower septal E/e’ ratio were observed on mitral annulus tissue Doppler. Mean left atrial volume was increased in both groups, but without any significant difference between them (Table 2).

**Table 2:** Demographic data

Variables	Normotensive patients (n = 31)	Hypertensive patients (n = 14)	p
**Measures**			
LVDD (mm)	4.56 ± 0.66	4.76 ± 0.60	0.3485
LVSD (cm)	2.42 ± 0.49	3.45 ± 0.46	0.0008
S/PW	2.03 ± 0.65	1.63 ± 0.44	0.0425
LA volume (ml/m^2^)	37.76 ± 17.14	38.97 ± 16.79	0.8245
RV (cm)	1.70 ± 0.43	1.71 ± 0.31	0.9757
EF% (Teichholz)	80.18 ± 5.76	74.01 ± 9.90	0.0116
E (cm/s)	78.23 ± 16.30	76.13 ± 26.83	0.7465
A (cm/s)	50.92 ± 16.92	80.70 ± 22.71	< 0.001
E/A	1.57 ± 0.56	0.96 ± 0.25	0.0003
EDT (ms)	241.90 ± 79.15	261.00 ± 66.23	0.4363
IVRT (ms)	119.94 ± 24.90	141.50 ± 35.08	0.0228
septal e’ (cm/s)	5.75 ± 1.30	4.43 ± 0.95	0.0015
lateral e’ (cm/s)	8.37 ± 2.79	7.21 ± 3.47	0.2386
Septal E/e’	13.98 ± 4.26	17.45 ± 6.21	0.0327
Lateral E/e’	10.18 ± 3.81	12.90 ± 6.81	0.0926
Mean E/e’	12.40 ± 3.73	15.71 ± 6.21	0.0696
**Diastolic dysfunction classification**			
Undetermined	41.9%	7.1%	0.0242
Grade 1	12.9%	50.0%	
Grade 2	41.9%	42.9%	
Grade 3	3.2%	0.0%	

Values expressed as mean±standard deviation or proportion, as indicated. LVDD: left ventricular diastolic diameter; LVSD: left ventricular systolic diameter; S/PW: interventricular septum/posterior wall ratio; LA: left atrium; RV: right ventricle; EF: ejection fraction; LVOT: left ventricular outflow tract; E: mitral flow E wave; A: mitral flow A wave; EDT: E wave deceleration time; IVRT: isovolumetric relaxation time; e’: mitral annulus tissue Doppler e’ wave.

In the hypertrophy type analysis, in the general sample, type III was the most frequent one (40%), followed by type II (31%), I (15.7%) and IV (13.3%), but no significant difference was observed between the groups regarding the type of hypertrophy. In this sample, there were no cases of concentric hypertrophy. Also, there was no significant difference between the groups regarding left ventricular outflow tract obstruction, with a higher percentage of the nonobstructive form in the overall sample (66.7%).

Mean blood pressure was higher in the hypertensive group. In this group, nine patients (64%) had increased blood pressure before the test, with 144x92 mmHg maximum. In the group without hypertension, six patients (19%) had a slight blood pressure increase with 135x84 mmHg maximum.

Regarding the medications used, more medications were used by the group of hypertensive patients, especially angiotensin receptor blockers, calcium antagonists and diuretics. No patient was taking cardiotoxic drugs or any drugs interfering with ventricular function.

## Discussion

The finding of significant reduction in myocardial strain in the hypertensive group suggests that these patients present greater impairment of ventricular function. Early detection of left ventricular dysfunction with preserved ejection fraction was only possible using the strain technique, not used in previous studies. Prior to the advent of strain, no significant abnormalities were observed in the comparison between these groups. In a study conducted in 1989 by Karan et al.,^17^ 78 patients diagnosed with HCM on echocardiography and cardiac catheterization were evaluated, including 39 hypertensive patients. The most relevant finding was higher hypertrophy in hypertensive patients, suggesting that SAH may increase hypertrophy in HCM. This study was important to define the existence of hypertrophic cardiomyopathy with hypertension, which was previously described as hypertensive hypertrophic cardiomyopathy.

In 1998, Dimitrow et al.^18^ published a study with 123 patients with HCM, 19.5% of whom were hypertensive, in which only functional class was evaluated. The study found that the association of SAH was more frequent in the elderly, but not rare in young people, which had worse functional class. In another study not using the strain technique, echocardiographic findings were similar between groups and SAH was also more frequent in the elderly.^19^

In 2014, Gonçalves et al.^20^ performed GLS analysis in a group of 229 pure hypertensive patients without HCM and preserved EF, and observed a reduction in GLS in 15.3% of the patients. However, no studies were found in the literature using the strain technique to compare HCM patients with and without associated SAH. In addition to detecting early changes in ventricular function, strain impairment may be a predictor of ventricular arrhythmia. In a publication with 400 HCM patients, those with GLS >-10% were four times more likely to have events than patients with GLS ≤-16%.^21^ Regional strain abnormalities in HCM may also be predictive of arrhythmia, as demonstrated by Correia et al.^22^ A study with 32 patients found mean septal strain >-10% with 89% sensitivity and 74% specificity for the development of non-sustained ventricular tachycardia, regardless of age or maximum wall thickness. These regional strain abnormalities may be related to areas with higher percentage of fibrosis on magnetic resonance imaging, being a potential substrate for the development of arrhythmias.^23,24^

Diastolic dysfunction was more evident in hypertensive patients in this sample. Hypertensive patients had a higher percentage of grade I or II dysfunction and a lower percentage of undetermined cases, according to the latest recommendations for diastolic function evaluation.^15^ It should be noted that left atrial volume, an important parameter in the assessment of diastolic function,^15,25,26^ was increased in both groups on average, with no significant difference between them. This means, in principle, that most patients had some degree of diastolic dysfunction, but it was better defined in hypertensive patients.

Regarding the type of hypertrophy, in the classification proposed by Maron et al.,^14^ which evaluated 125 patients, the most frequent type was type III (52%), followed by types II (20%), IV (18%) and I (10%). In another study, Reant et al.^27^ evaluated 271 patients using this classification, and the most frequently observed type was II (47%), followed by types III (35%), I (11%) and IV (7%). We have found a percentage similar to Maron’s classification regarding the most frequent types of hypertrophy, that is, types III and II, followed by types I and IV.

In the hypertensive group, mean age was higher, which may have influenced the evaluation of diastolic function and, perhaps, the strain analysis. Some studies have shown that myocardial strain presents a small reduction with age.^28,29^ Others have not observed a clear relationship between myocardial strain and age.^30,31^

We observed that mean blood pressure was higher in the hypertensive group, but there is no definition in the literature as to whether increased blood pressure at the time of the test may influence strain analysis.

### Study limitations

The strain technique requires regular heart rhythm, which limits its use in some situations, such as atrial fibrillation, which led to the exclusion of some patients. In the hypertensive group, mean age was higher and may have interfered with strain analysis and diastolic function analysis. Finally, long-term follow-up could provide further information about ventricular function behavior, since our study was cross-sectional.

## Conclusion

Patients with HCM and SAH had lower myocardial strain, suggesting greater impairment of left ventricular function, even with preserved ejection fraction. This finding may be related to a worse prognosis, with early evolution to heart failure and/or onset of ventricular arrhythmias. Prospective studies are needed to confirm this hypothesis.

## References

[1] Liew AC, Vassiliou VS, Cooper R, Raphael CE (2017). Hypertrophic cardiomyopathy- past, presente and future. J Clin Med.

[2] Brock R (1957). Functional obstruction of the left ventricle; acquired aortic subvalvar stenosis. Guys Hosp Rep.

[3] Teare D (1958). Asymmetrical hypertrophy of the heart in young adults. Br Heart J.

[4] Braunwald E, Aygen M.M (1963). Idiopathic myocardial hypertrophy without congestive heart failure or obstruction to blood flow: Clinical, hemodynamic and angiocardiographic studies in fourteen patients. Am J Med.

[5] Elliott PM, Anastasakis A, Borger MA, Borggrefe M, Cecchi F, Charron P (2014). 2014 ESC Guidelines on diagnosis and management of hypertrophic cardiomyopathy: the Task Force for the Diagnosis and Management of Hypertrophic Cardiomyopathy of the European Society of Cardiology (ESC). Eur Heart J.

[6] Albanesi Fº FM (1998). Cardiomyopathies. Arq. Bras. Cardiol..

[7] Bittencourt MI, Rocha RM, Albanesi Fº FM (2010). Hypertrophic cardiomyopathy. Arq. Bras. Cardiol.

[8] Malachias MVB, Jardim PCV, Almeida FA, Lima Júnior E, Feitosa GS (2016). 7th Brazilian Guideline of Arterial Hypertension: Pharmacological Treatment. Arq Bras Cardiol.

[9] Malta DC, Bernal RT, Andrade SS, Silva MMA, Velasquez-Melendez G (2017). Prevalence of and factors associated with self-reported high blood pressure in Brazilian adults. Rev Saúde Pública.

[10] Kato TS, Noda A, Izawa H, Yamada A, Obata K, Nagata K (2004). Discrimination of nonobstructive hypertrophic cardiomyopathy from hypertensive left ventricular hypertrophy on the basis of strain rate imaging by tissue Doppler ultrasonography. Circulation.

[11] Opdahl A, Helle-Valle T, Skulstad H, Smiseth OA (2015). Strain, strain rate, torsion, and twist: echocardiographic evaluation. Curr Cardiol Rep.

[12] Mirea O, Duchenne J, Voigt JU (2016). Recent advances in echocardiography: strain and strain rate imaging. F1000Res.

[13] To AC, Dhillon A, Desai MY (2011). Cardiac magnetic resonance in hypertrophic cardiomyopathy. JACC Cardiovasc Imaging.

[14] Lang RM, Badano LP, Mor-Avi V, Afilalo J, Armstrong A, Ernande L (2015). Recommendations for cardiac chamber quantification by echocardiography in adults: an update from the American Society of Echocardiography and the European Association of Cardiovascular Imaging. J Am Soc Echocardiogr.

[15] Maron BJ, Gottdiener JS, Epstein SE (1981). Patterns and significance of distribution of left ventricular hypertrophy in hypertrophic cardiomyopathy. A wide angle, two dimensional echocardiographic study of 125 patients. Am J Cardiol.

[16] Nagueh SF, Smiseth OA, Appleton CP, Byrd BF 3rd, Dokainish H, Edvardsen T (2016). Recommendations for the Evaluation of Left Ventricular Diastolic Function by echocardiography: An Update from the American Society of Echocardiography and the European Association of Cardiovascular Imaging. J Am Soc Echocardiogr.

[17] Karam R, Lever HM, Healy BP (1989). Hypertensive hypertrophic cardiomyopathy or hypertrophic cardiomyopathy with hypertension? A study of 78 patients. J Am Coll Cardiol.

[18] Dimitrow PP, Czarnecka D, Kawecka-Jaszcz K, Dubiel JS (1998). The frequency and functional impact of hypertension overlapping on hypertrophic cardiomyopathy:comparison between older and younger patients. J Hum Hypertens.

[19] Aslam F, Haque A, Foody J, Shirani J (2010). The frequency and functional impact of overlapping hypertension on hypertrophic cardiomyopathy: a single-center experience. J Clin Hypertens.

[20] Gonçalves S, Cortez-Dias N, Nunes A, Belo A, Zimbarra Cabrita I, Sousa C (2014). Left ventricular systolic dysfunction detected by speckle tracking in hypertensive patients with preserved ejection fraction. Rev Port Cardiol.

[21] Liu H, Pozios I, Haileselassie B, Nowbar A, Sorensen LL, Phillip S, Lu DY, Ventoulis I, Luo H, Abraham MR, Abraham TP (2017). Role of Global Longitudinal Strain in Predicting Outcomes in Hypertrophic Cardiomyopathy. Am J Cardiol.

[22] Correia E, Rodrigues B, Santos LF, Moreira D, Gama P, Cabral C (2011). Longitudinal left ventricular strain in hypertrophic cardiomyopathy: correlation with nonsustained ventricular tachycardia. Echocardiography.

[23] Funabashi N, Takaoka H, Horie S, Ozawa K, Daimon M, Takahashi M (2013). Regional peak longitudinal-strain by 2D speckle-tracking TTE provides useful informatio to distinguish fibrotic from non-fibrotic lesions in LV myocardium on cardiac MR in hypertrophic cardiomyopathy. Int J Cardiol.

[24] Funabashi N, Takaoka H, Ozawa K, Kamata T, Uehara M, Komuro I (2018). Quantitative Differentiation of LV Myocardium with and without Layer-Specific Fibrosis Using MRI in Hypertrophic Cardiomyopathy and Layer-Specific Strain TTE Analysis. Int Heart J.

[25] Hiemstra YL, Debonnaire P, Bootsma M, van Zwet EW, Delgado V, Schalij MJ (2017). Global Longitudinal Strain and Left Atrial Volume Index Provide ncremental Prognostic Value in Patients With Hypertrophic Cardiomyopathy. Circ Cardiovasc Imaging.

[26] Costabel JP, Galve E, Terricabras M, Ametrano C, Ronderos R, Baranchuk A, Evangelista A, Avegliano G (2018). E/e' ratio and left atrial area are predictors of atrial fibrillation in patients with hypertrophic cardiomyopathy. Echocardiography.

[27] Reant P, Donal E, Schnell F, Reynaud A, Daudin M, Pillois X (2015). Clinical and imaging description of the Maron subtypes of hypertrophic cardiomyopathy. Int J Cardiovasc Imaging.

[28] Alcidi GM, Esposito R, Evola V, Santoro C, Lembo M, Sorrentino R (2018). Normal reference values of multilayer longitudinal strain according to age decades in a healthy population: A single-centre experience. Eur Heart J Cardiovasc Imaging.

[29] Reckefuss N, Butz T, Horstkotte D, Faber L (2011). Evaluation of longitudinal and radial left ventricular function by two-dimensional speckle-tracking echocardiography in a large cohort of normal probands. Int J Cardiovasc Imaging.

[30] Menting ME, McGhie JS, Koopman LP, Vletter WB, Helbing WA, van den Bosch AE (2016). Normal myocardial strain values using 2D speckle tracking echocardiography in healthy adults aged 20 to 72 years. Echocardiography.

[31] Taylor RJ, Moody WE, Umar F, Edwards NC, Taylor TJ, Stegemann B (2015). Myocardial strain measurement with feature-tracking cardiovascular magnetic resonance: normal values. Eur Heart J Cardiovasc Imaging.

